# Phillyrin Mitigates Apoptosis and Oxidative Stress in Hydrogen Peroxide-Treated RPE Cells through Activation of the Nrf2 Signaling Pathway

**DOI:** 10.1155/2020/2684672

**Published:** 2020-10-12

**Authors:** Yuanyuan Du, Longtai You, Boran Ni, Na Sai, Wenping Wang, Mingyi Sun, Rui Xu, Yu Yao, Zhiqin Zhang, Changhai Qu, Xingbin Yin, Jian Ni

**Affiliations:** ^1^School of Chinese Materia Medica, Beijing University of Chinese Medicine, Beijing 100029, China; ^2^Dongzhimen Hospital Affiliated to Beijing University of Chinese Medicine, Beijing 100029, China; ^3^School of Pharmacy, Inner Mongolia Medical University, 010110 Hohhot, China; ^4^Beijing Research Institute of Chinese Medicine, Beijing University of Chinese Medicine, Beijing 100029, China

## Abstract

Oxidative stress-induced dysfunction or apoptosis in retinal pigment epithelial (RPE) cells is an important cause of dry age-related macular degeneration (AMD). Although phillyrin has been shown to exert significant antioxidant effects, the underlying mechanism of action remains unclear. The purpose of this study was to investigate the protective effect of phillyrin on hydrogen peroxide- (H_2_O_2-_) induced oxidative stress damage in RPE cells and the potential mechanism involved. It was found that phillyrin significantly protected RPE cells from H_2_O_2_ cytotoxicity. Furthermore, phillyrin alleviated oxidative stress-induced apoptosis via inhibition of endogenous and exogenous apoptotic pathways. Compared with the H_2_O_2_-treated group, the expressions of cleaved caspase-3, cleaved caspase-9, cleaved polymerase (PARP), death receptor Fas, and cleaved caspase-8, as well as Bax/Bcl-2 ratio were decreased in RPE cells after the phillyrin intervention. In addition, phillyrin reversed the oxidative stress-induced reductions in superoxide dismutase (SOD) and glutathione (GSH) levels and annulled the elevations in reactive oxygen species (ROS) and malondialdehyde (MDA), thereby restoring oxidant-antioxidant homeostasis. Phillyrin treatment upregulated the expressions of cyclin E, cyclin-dependent kinase 2 (CDK2), and cyclin A and downregulated the expressions of p21 and p-p53, thereby reversing the G0/G1 cell cycle arrest in H_2_O_2_-treated RPE cells. Pretreatment with phillyrin also increased the expressions of nuclear factor-erythroid 2-related factor 2 (Nrf2), total Nrf2, heme oxygenase-1 (HO-1), and NAD(P)H: quinone oxidoreductases-1 (NQO-1) in RPE cells and inhibited the formation of Kelch-like ECH-associated protein 1 (Keap1)/Nrf2 protein complex. Thus, phillyrin effectively protected RPE cells from oxidative stress through activation of the Nrf2 signaling pathway and inhibition of the mitochondria-dependent apoptosis pathway.

## 1. Introduction

Age-related macular degeneration (AMD), a degenerative disease that occurs in the center of the retina, causes irreversible vision loss in people over 65 years of age in developed countries. According to the World Health Organization (WHO) report, the incidence of AMD is 8.7%. In clinical practice, two forms of AMD are recognized: wet AMD and dry AMD, with dry AMD accounting for 90% of total AMD [1]. The molecular mechanism underlying wet AMD is closely related to choroidal neovascularization. Currently, vascular endothelial growth factor (VEGF) antagonists are drugs of first choice in the treatment of wet AMD, and their effects are significant [2]. Dry AMD involves advanced forms of RPE and atrophy of photoreceptor cells [3]. Various risk factors such as age, smoking, obesity, and drinking induce AMD [4, 5]. Currently, there are no specific therapeutic drugs for dry AMD. However, a growing number of studies have shown that protection of retinal mitochondrial membrane from oxidative stress is a viable option for the treatment of dry AMD [6–9].

Oxidative stress leads to RPE cell dysfunction or apoptosis, and it is an important factor in the pathology of AMD [10]. External factors such as cigarette smoking, exposure to blue light, high concentrations of unsaturated fatty acids, and high metabolic activity lead to excessive ROS production in RPE cells, resulting in cell dysfunction or apoptosis [11, 12]. Under normal conditions, Nrf2 binds to Keap1 in the cytoplasm and is not actively transported into the nucleus. However, when the levels of ROS increase, Nrf2 is stimulated, and its binding to Keap1 becomes unstable, resulting in its release and transfer to the nucleus [13]. Antioxidant response element (ARE) is a *cis*-acting element [14]. In the nucleus, Nrf2 binds to ARE and transcribes downstream genes through related activation procedures, thereby activating the expression of the oxidoreductase *λ*-glutamyl cysteine synthetase catalytic subunit (GCLC)/HO-1/NQO-1 which exhibits antioxidant effects [15]. Therefore, the Nrf2 pathway is considered an effective pathway for regulating ROS and enabling cells to reduce damage due to oxidative stress. This study investigated the protective effect of phillyrin against oxidative stress-induced damage in RPE cells and the underlying mechanism.

Under oxidative stress, ROS are produced in large amounts in cells [16]. Studies have shown that under normal circumstances, intracellular ROS arise from the activity of oxidase, whereas under pathological conditions, the mitochondrial respiratory chain is an important pathway for ROS [17]. Cytochrome *c* is an important member of the mitochondrial respiratory chain. It is located on the outer side of the mitochondrial inner membrane, and it cannot enter the cytoplasm freely [18]. When the amount of ROS in the cell is elevated, there is enhancement of lipid peroxidation which destroys the mitochondrial inner membrane containing unsaturated fatty acids, causing release of large amounts of cytochrome *c* which are transferred to the cytoplasm. At the same time, the ROS interact with Bax and promote cytochrome *c* release into the cytoplasm [19]. Caspases are important proteins involved in regulation of apoptosis [20]. In the cytoplasm, cytochrome *c* combines with caspases-9 to form an apoptotic body [21, 22]. In turn, the apoptotic body activates downstream caspase-3, enters the final pathway of endogenous and exogenous apoptosis pathways, and eventually leads to apoptosis [23, 24]. In this study, phillyrin was used to protect RPE cells from oxidative stress damage by inhibiting the mitochondrial-dependent apoptosis pathway.

**Figure 1:**
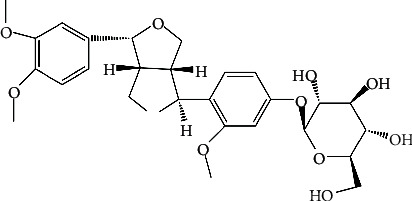
Chemical structure of phillyrin.

Phillyrin (Figure 1) was obtained from an extract of the dried fruit of *Forsythia suspensa (Thunb.) Vah*, a Chinese traditional herbaceous plant. Studies have shown that phillyrin exerts antibacterial, antiviral, anti-inflammatory, antioxidant, and other pharmacological effects [25]. Another study showed that phillyrin exerts protective effect against H_2_O_2_-induced oxidative stress damage and PC12 cell apoptosis, indicating that it possesses significant antioxidant properties [26, 27]. However, it is not clear whether phillyrin protects RPE cells from H_2_O_2_-induced oxidative stress and the possible underlying mechanism. This study was aimed at providing a new therapeutic strategy for the treatment of AMD by investigating the protective effect of phillyrin against H_2_O_2_-induced oxidative stress injury in RPE cells and the mechanism involved.

## 2. Materials and Methods

### 2.1. Reagents and Chemicals

Human ARPE-19 cell line was purchased from Beijing Beina Chuanglian Biotechnology Research Institute (Beijing, China). Phillyrin (batch number Z17A8X34077, purity > 98%) was a product of the China Food and Drug Administration (Beijing, China). Dulbecco's Modified Eagle's Medium/nutrient mixture F-12 (DMEM/F-12), fetal bovine serum (FBS), penicillin, and streptomycin solutions were purchased from Corning (NY, USA). Trypsin (0.05%) and phosphate buffered saline (PBS) were produced by Gibco, Invitrogen (Carlsbad, CA, USA). Dimethyl sulfoxide (DMSO), 3-(4,5-dimethylthiazol-2-yl-)-2,5-diphenyltetrazolium bromide (MTT), ML385, N-acetylcysteine (NAC)m and H_2_O_2_ solution were purchased from Sigma-Aldrich (St. Louis, MO, USA). Antibodies against Fas (ab82419), cytochrome *c* (ab90529), cleaved caspase-3 (ab2302), cleaved caspase-9 (ab2324), NQO1 (ab80588), Keap1 (ab118285), Bcl-2 (ab185002), Nrf2 (ab62352), CDK2 (ab32147), cyclin A (ab33911), cyclin E (ab181591), Bax (ab53154), *β*-actin (ab8226), p21 (ab188224), Histone H3 (ab1791), COX IV (ab16056) p53 (ab241556), pro caspase-3, pro caspase-8, and pro caspase-9 were obtained from Abcam, Cambridge, (MA, USA). Antibodies against p-p53 (9286S), cleaved caspase-8 (9496S), cleaved PARP (5625S), and HO-1 (86806S) were purchased from Cell Signaling Technology, Beverly, (MA, USA).

### 2.2. Cell Cultures

**Figure 2:**
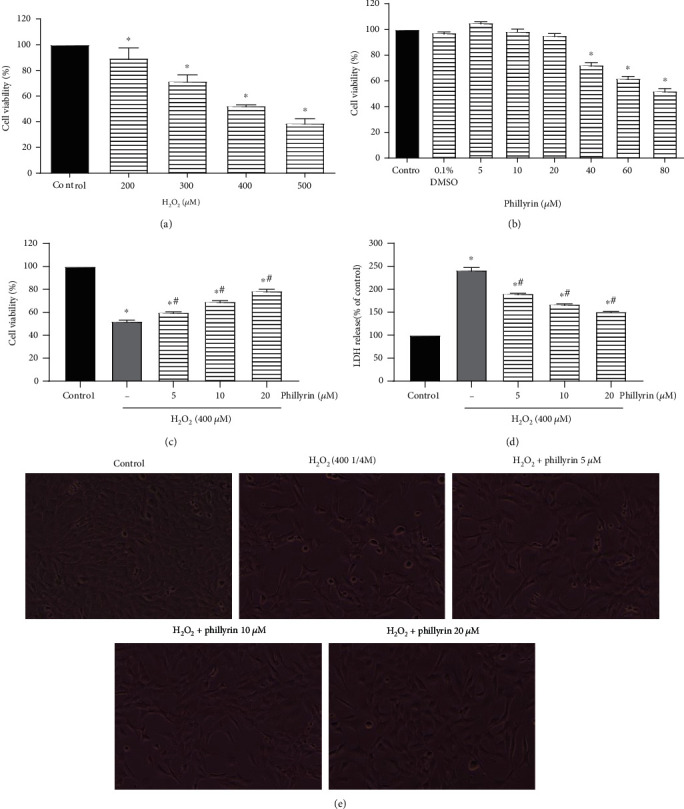
Protective effect of phillyrin on H_2_O_2_-induced cytotoxicity in RPEs. (a) RPE cells were treated with indicated concentrations of H_2_O_2_ for 6 hours, and cell viability was analyzed with the MTT assay. (b) Cell viability of RPE cells treated with different concentrations of phillyrin. (c) RPE cells were pretreated under different concentrations of phillyrin (5, 10, and 20 *μ*M) for 24 hours and then incubated with 400 *μ*M H_2_O_2_ for 6 hours. The cytoprotective effect of phillyrin was measured with the MTT assay. (d) LDH profile showing the dose-dependent cytoprotective effect of phillyrin. (e) Morphological changes in RPE cells (original magnification: ×200). Compared with the H_2_O_2_ group, phillyrin pretreatment protected RPE cells in a dose-dependent manner. Data are expressed as mean ± S.D. Experiments were repeated three times (^∗^*p* < 0.05 vs. control, ^#^*p* < 0.05 vs. H_2_O_2_-treated group).

Human RPE cell line (HRPE-19) was maintained in DMEM/F12 containing 10% FBS and 1% penicillin/streptomycin in a 5% CO_2_ humidified incubator at 37°C. Phillyrin was dissolved in DMSO and preserved at -20°C. The final concentration of DMSO in the culture medium was kept below 0.1%, a concentration which has been shown to be nonlethal to cells. As shown in Figure 2(b), when the cells reached about 80% confluence, cells in the H_2_O_2_ group were treated with only 400 *μ*M H_2_O_2_ for 6 hours. In contrast, cells in the phillyrin+H_2_O_2_ groups were pretreated with 5, 10, or 20 *μ*M phillyrin for 24 hours, prior to exposure to 400 *μ*M H_2_O_2_ for another 6 hours. Logarithmic growth phase cells were used in this study.

### 2.3. Cell Viability Assay and Morphology Examination

The MTT analysis was used to determine the effect of phillyrin on the viability of RPE cells. The cells were inoculated in 96-well plates at a density of 5 × 10^3^ cells/well overnight. Cells in the pretreatment groups were treated with medium containing 5-20 *μ*M phillyrin, followed by incubation for 24 hours. Thereafter, 400 *μ*M H_2_O_2_ was added to the cells, and further incubation was carried out for 6 hours. Then, 100 *μ*L of 5 mg/mL MTT solution was added to the wells, followed by incubation for 4 hours. Finally, the medium was discarded, and the formazan crystals formed were dissolved in 150 *μ*L of 0.1% DMSO, shaken at 50 times/min for 10 minutes, and the absorbance of each formazan solution was read at 490 nm in a microplate reader (Thermo Fisher Scientific, NYC, USA). The morphological changes in the treated cells were observed under an inverted Olympus IX71 fluorescence microscope (Olympus Corporation, Tokyo, Japan) and photographed.

### 2.4. Determination of the LDH Activity

The cells were seeded into 96-well plates at a density of 5 × 10^3^ cells/well, and incubation was carried out as described in Cell Cultures. Following tryptic digestion, the cell suspension was centrifuged at 3000 rpm for 10 minutes. The supernatant was subjected to assay of the lactate dehydrogenase (LDH) activity using the LDH assay kit (Beyotime Biotechnology, Shanghai, China) according to the manufacturer's instructions.

### 2.5. TUNEL Staining for Determination of Apoptosis

The RPE cells were seeded in a 6-well plate at a density of 2 × 10^5^ cells/well. After establishing the model, the cells in each group were fixed with paraformaldehyde solution, and cell apoptosis was determined using TdT-mediated dUTP Nick-End Labeling (TUNEL) analysis kits (Beyotime Biotechnology, Shanghai, China) as per the manufacturer's protocol. The nuclei of positive cells (i.e., apoptotic cells) were stained brownish yellow. The degree of apoptosis in each group was noted and photographed under a fluorescence microscope.

### 2.6. Apoptosis Analysis

The cells were seeded in 6-well plates at a density of 2 × 10^5^ cells/well and cocultured with graded concentrations of phillyrin (5, 10 and 20 *μ*M) for 24 hours. Then, 400 *μ*M H_2_O_2_ was added to the cells, followed by incubation for 6 hours. The cells were washed with PBS and thoroughly mixed with 295 *μ*L of binding buffer, in line with the instructions of apoptosis kits (Beyotime Biotechnology, Shanghai, China). Subsequently, the cells were stained with 5 *μ*L of Annexin V-fluorescein isothiocyanate (FITC) and 10 *μ*L of propidium iodide (PI) and left in the dark at room temperature for 25 minutes. Apoptosis was assessed using flow cytometry (BD Biosciences, New Jersey, USA).

### 2.7. Measurement of Intracellular ROS

The ROS level in RPE cells was measured using the 2,7-dichlorofluorescin diacetate (DCFH-DA) assay. This is based on the principle that DCFH-DA can pass freely through the cell membrane and be hydrolyzed by esterase to generate DCFH. Active oxygen in the cell oxidizes nonfluorescent DCFH to generate fluorescent DCF. Thus, the degree of DCF fluorescence is an index of the level of active oxygen in the cell [28]. In this study, RPE cells were seeded in a 6-well plate at a density of 2 × 10^5^ cells/well and incubated as described in section Cell Cultures. Then, the cells were incubated with a 10 mM DCFH-DA working solution at 37°C for 30 minutes in the dark. The cells were then processed according to instructions on kits (Beyotime Biotechnology,Shanghai, China), and the percentage of fluorescent positive cells was quantitatively analyzed flow cytometrically.

### 2.8. Determination of Cellular Oxidative Status

As usual, the RPE cells were seeded in 6-well plates at a density of 2 × 10^5^ cells/well, and the cells were incubated as described in section Cell Cultures. After discarding the medium in each well, the cells were collected and centrifuged at 12000 rpm for 10 minutes. The resultant supernatant was discarded, and the cells were lysed on an ice table using the radio-immunoprecipitation assay (RIPA) buffer. The protein content of each lysate was quantified with the bicinchoninic acid (BCA) protein quantification kit (Beyotime Biotechnology, Shanghai, China). The levels of malondialdehyde (MDA), GSH, and SOD in the cells were determined strictly in accordance with the instructions of the respective assay kits (Beyotime Biotechnology, Shanghai, China). The final result was expressed based on the content of target protein relative to total protein.

### 2.9. Determination of the Effect of Phillyrin on MMP

Mitochondrial membrane potential (MMP) is measured with the fluorescent probe 5,5′,6,6′-tetrachloro-1,1′,3,3′-tetraethyl-imidacarbocyanine iodide (JC-1). When JC-1 accumulates in the mitochondrial matrix, it produces red fluorescence, indicating that the mitochondrial membrane potential is high. In contrast, a decrease in mitochondrial membrane potential is reflected in reduced red fluorescence. Thus, changes in mitochondrial membrane potential can be judged based on changes in fluorescence [29]. The cells were cultured as indicated in Cell Viability and Morphology Examination. After discarding the supernatant, the cells were washed with PBS, and 1 mL of cell culture medium and 1 mL of JC-1 staining working solution were thoroughly mixed and added to 6-well plates. Then, the cells were incubated in a cell incubator at 37°C for 20 minutes, followed by washing 3 times with 1 mL of JC-1 staining buffer. After discarding the supernatant, the cells were added to 2 mL of culture medium and observed under a fluorescence microscope. The fluorescence pictures were analyzed using ImageJ image processing software, and the fluorescence intensity of cells in each group was determined.

### 2.10. Cell Cycle Analysis

Cell cycle analysis was carried out in strict accordance with the instructions of the cell cycle kit (Beyotime Biotechnology, Shanghai, China). The RPE cells were seeded in 6-well plates at a density of 2 × 10^5^ cells/well and incubated as described in Cell Viability and Morphology Examination. The cell culture medium was discarded, and the cells were digested with trypsin and washed twice with PBS to make a single cell suspension which was fixed overnight in 75% ethanol at 4°C. Then, the ethanol was discarded, and the cells were washed twice with PBS, followed by suspension of the cells in 0.5 mL of staining buffer containing 10 *μ*L propidium iodide (PI) and 10 *μ*L ribonuclease (RNase). The cells were incubated for 30 minutes in the dark, after which the cell cycle was analyzed with flow cytometry.

### 2.11. Western Blotting

As usual, RPE cells were inoculated into 6-well plates at a density of 2 × 10^5^ cells/well. After treatment as per Cell Viability Assay and Morphology Examination, the cells in each well were lysed for 30 minutes on ice by addition of 100 *μ*L ice-cold RIPA cell lysis buffer (Sigma, St. Louis, MO, USA) containing 1 mM phenylmethylsulfonyl fluoride (PMSF). Thereafter, the lysates were centrifuged at 4°C at 12000 rpm for 15 minutes, and the protein contents of the supernatants were measured. Then, equal amounts of protein (50-70 *μ*g) were subjected to sodium dodecyl sulfate-polyacrylamide gel electrophoresis (SDS-PAGE), followed by transfer to polyvinylidene fluoride (PVDF) membrane. The membrane was blocked by incubation with 5% skim milk powder at 37°C for 1 h, prior to incubation overnight at 4°C with diluted primary antibodies against Fas, cytochrome *c*, cleaved caspase-3, cleaved caspase-9, NQO1, p53, Keap1, Bcl-2, Nrf2, CDK2, cyclin A, cyclin E, Bax, p21, p-p53, cleaved caspase-8, cleaved PARP, and HO-1. This was followed by incubation with a suitable secondary antibody at room temperature for 1 hour. Color development was done with chemiluminescence, while the gel imaging system was used for quantitative image analysis. Histone H3, COX IV, and *β*-actin were used as internal controls.

### 2.12. Immunoprecipitation

The RPE cells were treated as indicated in Cell Viability Assay and Morphology Examination and lysed with RIPA buffer containing protease inhibitors so as to maintain the integrity of the Keap1/Nrf2 protein complex. The whole cell lysate was incubated overnight at 4°C with the corresponding primary antibody, with shaking. The protein A/G magnetic beads were washed 3 times with appropriate amount of lysis buffer and centrifuged at 3000 rpm for 3 minutes. The cell lysate was mixed with 10 *μ*L of the pretreated protein A/G magnetic beads and then slowly incubated at 4°C for 4 hours to achieve binding of the antibody to the protein A/G magnetic beads. Anti-Keep1 antibody (2 *μ*g) was added to whole cell lysate and incubated at 4°C overnight. After washing the immunoprecipitated complex 5 times with RIPA buffer, it was boiled in SDS sample buffer for 5 minutes. Lastly, the immunoblots of the Nrf2 antibody were obtained using SDS-PAGE electrophoresis.

### 2.13. Statistical Analysis

All data were expressed as mean ± S.D. of at least three independent experiments. Statistical analysis was performed using GraphPad Prism 8.0 statistical software. Statistical differences were determined with one-way analysis of variance and LSD test. Values *p* < 0.05 were considered statistically significant.

## 3. Results

### 3.1. Phillyrin Protected RPE Cells from H_2_O_2_-Induced Cytotoxicity

First, the cultured RPE cells were treated with different concentrations of H_2_O_2_ (200-500 *μ*M) for 6 hours to obtain suitable oxidative stress conditions. Then, cell viability was determined using the MTT assay. As shown in Figure 2(a), when the H_2_O_2_ concentration was 400 *μ*M, 50% of RPE viability was lost. Therefore, treatment with 400 *μ*M H_2_O_2_ for 6 hours was selected as the condition for subsequent experiments. Next, in order to find the noncytotoxic range of phillyrin to RPE cells, the cells were incubated with different concentrations of phillyrin (5-80 *μ*M) for 24 hours. As shown in Figure 2(b), 40 *μ*M phillyrin was cytotoxic. Thus, phillyrin doses of 5, 10 ,and 20 *μ*M were used in subsequent experiments. Finally, after incubating the RPE cells with 5, 10, and 20 *μ*M phillyrin for 24 hours, the cells were treated with H_2_O_2_ for 6 hours. As shown in Figure 2(c), 20 *μ*M phillyrin significantly increased the viability of RPE cells, when compared with the H_2_O_2_-treated group. At the same time, the results were consistent with those of LDH shown in Figure 2(d). Morphological pictures confirmed the protective effect of phillyrin, as shown in Figure 2(e). Compared with the H_2_O_2_ group, phillyrin pretreatment protected RPE cells in a dose-dependent manner.

### 3.2. Phillyrin Protected RPE Cells from H_2_O_2_-Induced Apoptosis

**Figure 3:**
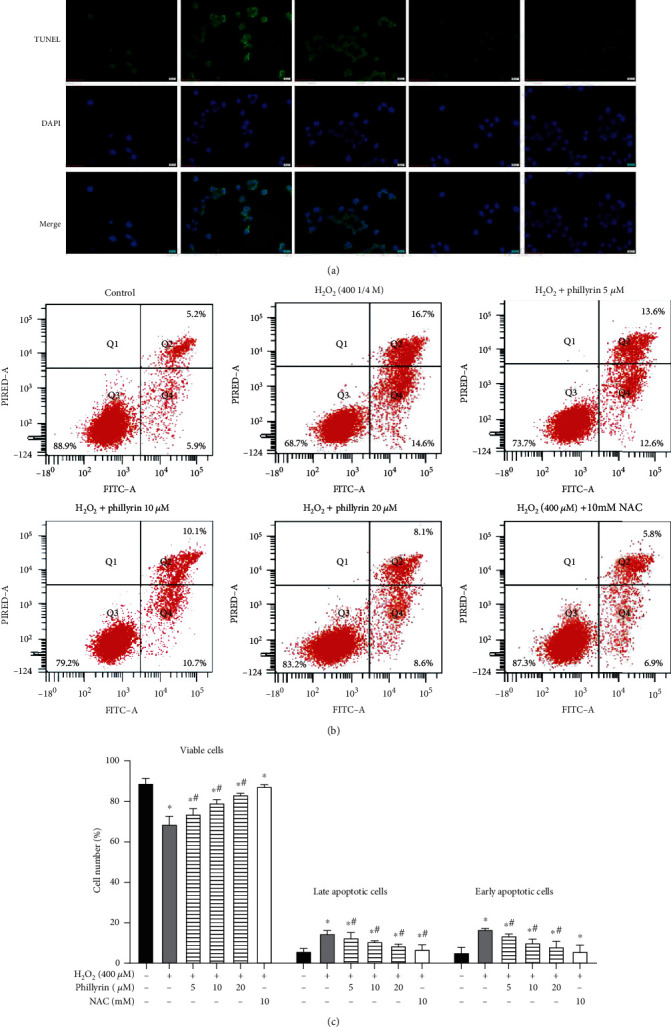
Protective effect of phillyrin on apoptosis of RPE cells. (a) Morphological changes in the nuclei of RPE cells, as revealed via TUNEL staining. (b) Apoptosis of RPE cells treated with different concentrations of phillyrin, as detected using double staining of Annexin V-FITC/PI and flow cytometry. (c) Histogram of average cell fluorescence showing survival, early apoptotic, and late apoptotic cells. Data are expressed as mean ± S.D. Experiments were repeated three times (^∗^*p* < 0.05 vs. control, ^#^*p* < 0.05 vs. H_2_O_2_-treated group).

In order to fully establish the protective effect of phillyrin on H_2_O_2_-induced apoptosis of RPE cells, two different methods were used: Annexin V-FITC/PI flow cytometry and TUNEL nuclear staining. As shown in Figure 3(a), cells in the normal and phillyrin-pretreated groups had uniform blue chromatin and normal nuclear morphologies. In contrast, cells in the H_2_O_2_-treated group had concentrated RPE nuclei, enhanced fluorescence, and fragmented chromatin. However, phillyrin treatment effectively reduced the nuclear debris and nuclear concentration and showed a dose-dependent protection. As shown in Figures 3(b) and 3(c), flow cytometry experiments further confirmed that phillyrin markedly inhibited H_2_O_2_-induced apoptosis in RPE cells. The Annexin V-positive cells in the H_2_O_2_-treated group were significantly increased, when compared with the control group, but this change was significantly reversed after phillyrin pretreatment. In all, these results indicate that phillyrin effectively inhibited H_2_O_2_-induced apoptosis in RPE cells.

### 3.3. Phillyrin Protected RPE Cells from H_2_O_2_-Induced Oxidative Stress and Mitochondrial Dysfunction

#### 3.3.1. Phillyrin Protected RPE Cells from H_2_O_2_-Induced Oxidative Stress

**Figure 4:**
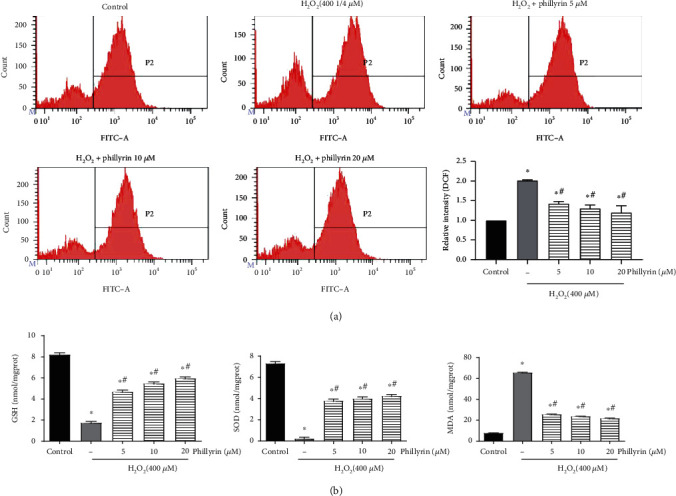
Effect of phillyrin on ROS and the antioxidant enzyme activity in RPE cells after oxidative stress injury. (a) Effect of H_2_O_2_ and different concentrations of phillyrin on ROS, as evaluated using flow cytometry. (b) Effect of H_2_O_2_ and different concentrations of phillyrin on the MDA content and levels of antioxidants SOD and GSH in the cells. Data are expressed as mean ± S.D. Experiments were repeated three times (^∗^*p* < 0.05 vs. control, ^#^*p* < 0.05 vs. H_2_O_2_-treated group).

The level of intracellular ROS affects oxidative stress and apoptosis [30]. High ROS and MDA levels indicate high oxidation levels, while high SOD and GSH levels reflect high antioxidant levels [31]. As shown in Figures 4(a) and 4(b), compared with the control group, H_2_O_2_ increased intracellular ROS and MDA levels, while it decreased SOD and GSH levels. In contrast, after phillyrin treatment, ROS and MDA levels were decreased, while SOD and GSH levels were increased. These results show that phillyrin effectively inhibited oxidative stress in RPE cells. In addition, as shown in Figure 3(c), pretreatment with NAC significantly reduced apoptosis of RPE cells, further proving that excessive intracellular ROS production is related to oxidative stress-induced apoptosis. Therefore, phillyrin protected RPE cells by inhibiting oxidative stress-mediated apoptosis.

#### 3.3.2. Phillyrin Protected RPEs from H_2_O_2_-Induced Mitochondrial Dysfunction

**Figure 5:**
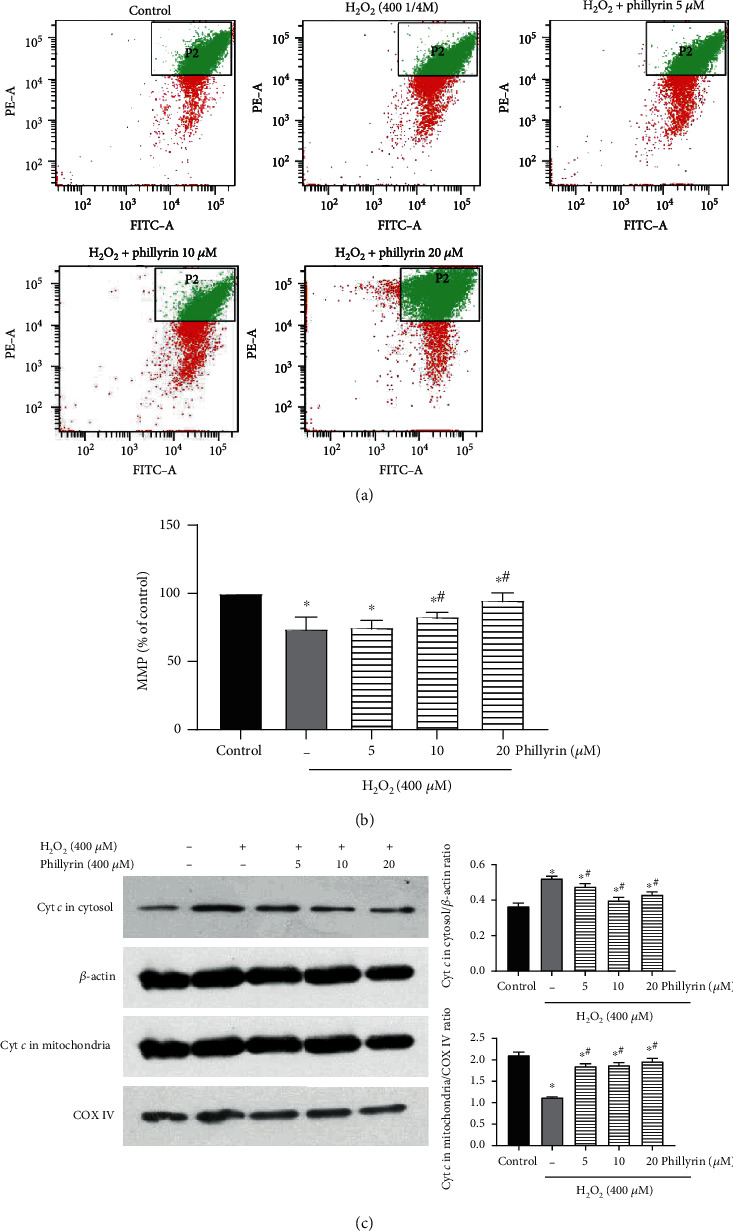
Effect of phillyrin on MMP in H_2_O_2_-treated RPE cells. (a) MMP in the cells after intervention with H_2_O_2_ and different concentrations of phillyrin, as determined using JC-1 staining. (b) Histogram of average cell fluorescence. (c) Effect of different concentrations of phillyrin on the release of cytochrome *c* in mitochondria and cytoplasm, as measured using Western blotting. The protein bands were quantified with density analysis and statistically analyzed. The internal controls for cytoplasm and mitochondria were *β*-actin and COX IV, respectively. Data are expressed as mean ± S.D. Experiments were repeated three times (^∗^*p* < 0.05 vs. control, ^#^*p* < 0.05 vs. H_2_O_2_-treated group).

**Figure 6:**
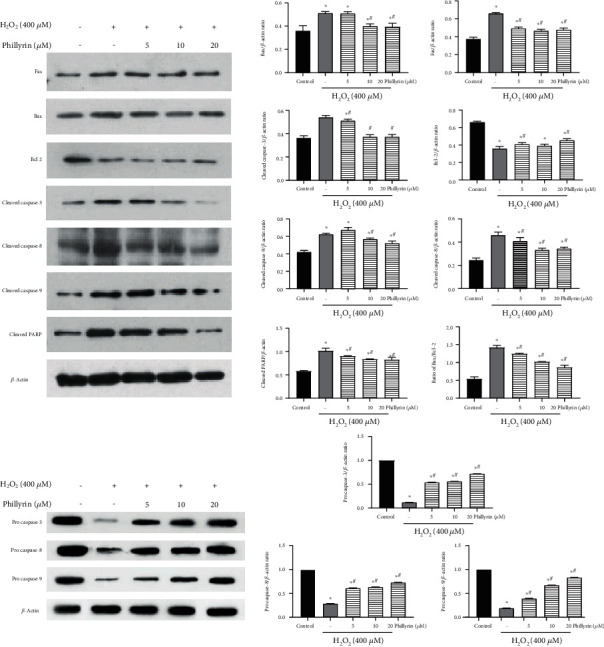
Effect of phillyrin on the expressions of apoptosis-related proteins in H_2_O_2_-treated RPE cells. Data are expressed as mean ± S.D. Experiments were repeated three times (^∗^*p* < 0.05 vs. control, ^#^*p* < 0.05 vs. H_2_O_2_-treated group).

Previous experiments have demonstrated that phillyrin effectively inhibited H_2_O_2_-induced apoptosis of RPE cells. Next, the link between H_2_O_2_-induced apoptosis and mitochondrial dysfunction was investigated. When MMP is impaired, the cytochrome *c* released into the cytoplasm from the mitochondria activates cell apoptosis [32]. As shown in Figures 5(a) and 5(b), compared with the control group, H_2_O_2_ significantly decreased MMP, but phillyrin treatment of the H_2_O_2_ groups effectively increased MMP in a dose-dependent manner. The apoptosis-regulating proteins Bcl-2 and Bax mediate the mitochondrial-dependent apoptosis pathway [33]. As shown in Figure 6, H_2_O_2_ increased the Bax/Bcl-2 ratio, while in the phillyrin+H_2_O_2_ groups, the Bax expression was decreased while the Bcl-2 expression was upregulated. At the same time, as shown in Figure 5(c), H_2_O_2_ released cytochrome *c* from the mitochondria into the cytoplasm, while in the phillyrin+H_2_O_2_ groups, cytochrome *c* release from the mitochondria was significantly inhibited in a dose-dependent manner. Therefore, phillyrin effectively inhibited the mitochondrial dysfunction pathway apoptosis induced by H_2_O_2_.

### 3.4. Phillyrin Effectively Reversed H_2_O_2_-Induced Cell Cycle Arrest of RPE Cells in the G0/G1 Phase

**Figure 7:**
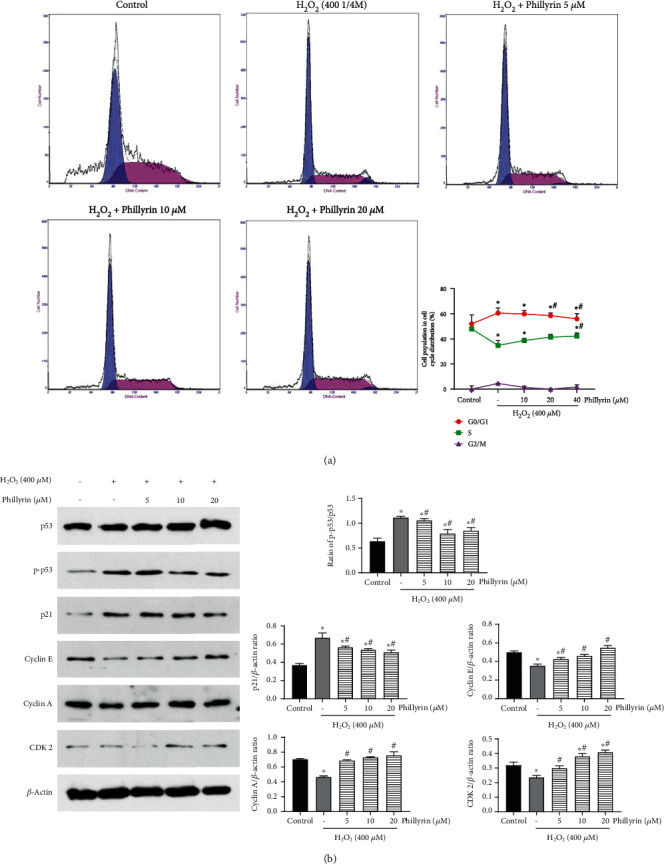
Effect of phillyrin on H_2_O_2_-indued cell cycle arrets in RPE cells. (a) Effect of different concentrations of phillyrin on each stage of the cell cycle. (b) Effect of phillyrin on the expression levels of cell cycle-related proteins, as measured using Western blotting. Data are expressed as mean ± S.D. Experiments were repeated three times (^∗^*p* < 0.05 vs. control, ^#^*p* < 0.05 vs. H_2_O_2_-treated group).

Studies have reported that cell cycle arrest is closely related to apoptosis [34]. To confirm whether phillyrin protected RPE cells by affecting the cell cycle, the cell cycle distribution was analyzed flow cytometrically after phillyrin treatment. As shown in Figure 7(a), H_2_O_2_ caused decrease in population of RPE cells in S-phase, while the number of RPE cells in the G0/G1 phase was increased. However, phillyrin treatment significantly increased the number of S-phase cells, while the G0/G1 phase cell population was decreased. These results indicate that H_2_O_2_ arrested the cell cycle at the G0/G1 phase, while phillyrin reversed the situation. In addition, as shown in Figure 7(b), cells treated with H_2_O_2_ alone had significantly upregulated protein expression levels of p53, p-p53, and p21. However, phillyrin pretreatment reversed the expression levels of these proteins in a dose-dependent manner. Cyclin-dependent kinases (CDKs) are involved in the cell cycle [35]. As shown in Figure 7(b), treatment of RPE cells with H_2_O_2_ alone resulted in significant downregulations of the expressions of cyclin E, CDK2, and cyclin A. However, phillyrin treatment resulted in significant and dose-dependent increases in the expressions of cyclin E, CDK2, and cyclin A. Taken together, these results indicate that phillyrin effectively inhibited H_2_O_2_-induced cell cycle arrest in RPE cells.

### 3.5. Phillyrin Regulated the Expressions of Apoptosis-Related Proteins in H_2_O_2_-Treated RPE Cells

Studies have shown that apoptosis involves endogenous and exogenous pathways. Activation of associated death receptors leads to exogenous apoptosis, while the endogenous pathway refers to mitochondria-induced apoptosis [36]. Both pathways directly activate the downstream caspases [37]. This study determined the mechanism involved in the protective effect of phillyrin by assaying the expressions of apoptosis-related proteins. As shown in Figure 6, the protein expressions of cleaved caspase-8, cleaved caspase-9, cleaved caspase-3, and cleaved PARP in cells treated with H_2_O_2_ only were significantly higher than their corresponding expressions after phillyrin treatment. Phillyrin downregulated the expressions of these proteins in a dose-dependent manner. In addition, Figure 6 shows that cells treated with H_2_O_2_ alone had significantly increased protein expressions of Fas (a typical death receptor) and Bax, when compared to untreated cells. After intervention with phillyrin, the expressions of Fas and Bax were effectively suppressed in a dose-dependent manner, while the expressions of pro caspases-8, -9, and -3 were increased in a dose-dependent manner. These results indicate that phillyrin effectively inhibited the H_2_O_2_-induced activation of the death receptor apoptotic pathway and mitochondria-dependent apoptotic pathway in RPE cells.

### 3.6. Phillyrin Promoted Activation of the Nrf2 Pathway and Inhibited Oxidative Stress

**Figure 8:**
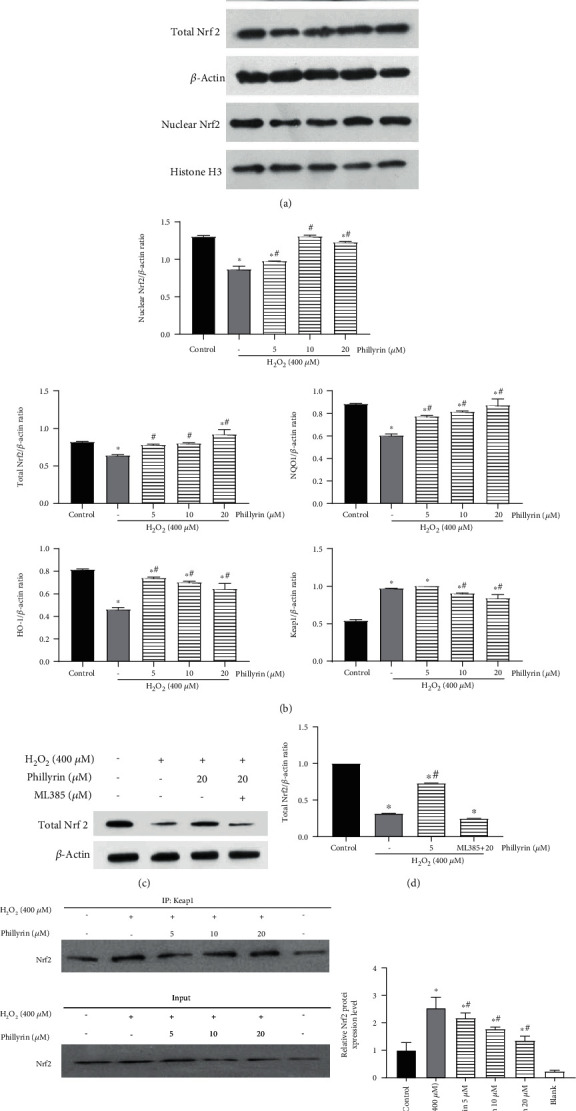
Effect of phillyrin on the Nrf2/HO-1 pathway in H_2_O_2_-treated RPE cells. (a) Expression levels of HO-1, NQO1, Keap1, total Nrf2, and nuclear Nrf2, as assayed using Western blotting. (b) Quantitative analysis of the protein bands using optical density analysis. (c) After adding ML385 to the high-dose phillyrin+H_2_O_2_ group, the expression levels of total Nrf2, as assayed using Western blotting. (d) Quantitative analysis of the protein bands using optical density analysis. (e) Effect of phillyrin on the formation of the Keap1/Nrf2 complex, as detected using the coimmunoprecipitation assay. Data are expressed as mean ± S.D. Experiments were repeated three times (^∗^*p* < 0.05 vs. control, ^#^*p* < 0.05 vs. H_2_O_2_-treated group).

In Figures 8(a) and 8(b), compared with control group, the Keap1 protein expression was increased in RPE cells treated with H_2_O_2_ alone, while the expressions of total Nrf2 and nuclear Nrf2 were significantly decreased. At the same time, the expressions of downstream oxidoreductases (HO-1 and NQO1) were downregulated, while the level of the Keap1/Nrf2 protein complex was increased (Figure 8(e)). However, pretreatment with phillyrin significantly inhibited the expression of Keap1 and promoted the expressions of total Nrf2 and nuclear Nrf2 in a concentration-dependent manner. Furthermore, the expressions of HO-1 and NQO1 were significantly upregulated, while the formation of the Keap1/Nrf2 protein complex was blocked (Figure 8(e)). It has been reported that ML385 is an inhibitor of Nrf2 [38]. In order to investigate if the protective effect of phillyrin occurred via activation of the Nrf2 signaling pathway, ML385 was added to the phillyrin intervention group. The results revealed that the expression of total Nrf2 did not increase significantly (Figures 8(c) and 8(d)). These results show that H_2_O_2_ disrupted the oxidation-antioxidation balance in RPE cells, leading to the inactivation of Nrf2 antioxidant pathway, while phillyrin pretreatment effectively protected the oxidation-antioxidant balance of RPEs by activating the antioxidant pathway of Keap1/Nrf2/ARE.

## 4. Discussion

Phillyrin is a natural diepoxylignan compound extracted from *Forsythia suspensa (Thunb.) Vah.* It has a wide range of pharmacological properties such as anti-inflammatory, antioxidation, and antiendotoxin effects [39]. Phillyrin produces good results in the clinical treatment of dry AMD, although its underlying mechanism has not been fully elucidated [40–42].

Studies have shown that apoptosis of RPE cells is an important factor leading to the development of dry AMD [43]. The results of MTT and LDH assays showed that H_2_O_2_ exerted significant cytotoxic effect on RPE cells. However, the viability of RPE cells was significantly increased by phillyrin. Moreover, phillyrin had the cytoprotective effect on RPE cells, as was evident from results of morphological examination. Therefore, it can be reasonably inferred that phillyrin protects RPE cells from H_2_O_2_-cytotoxicity in a dose-dependent manner.

**Figure 9:**
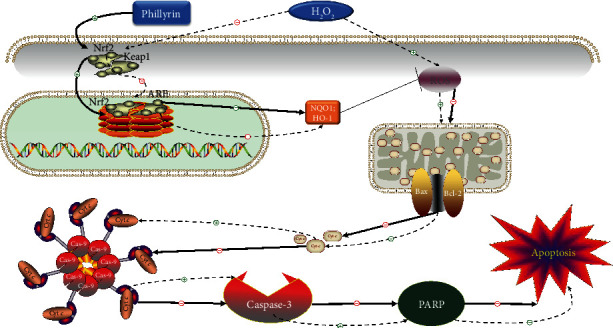
Schematic diagram of the mechanism of forsythin protecting RPE cells from apoptosis induced by hydrogen peroxide through the Nrf2 signaling pathway.

Apoptosis is a programmed death process that is precisely regulated by a series of signal molecules. The signal transduction of apoptosis proceeds through two basic pathways: the exogenous pathway and the endogenous pathway. The exogenous route is mediated by death receptors, e.g., Fas [44]. In contrast, the endogenous pathway is mediated by increased permeability of the mitochondrial outer membrane [45]. In the exogenous pathway of apoptosis, Fas-associated death domain (FADD) combines with pro caspase-8 to form a death-inducing signaling complex (DISC). Pro caspase-8 is homologous activated to caspase-8. Then, activated caspase-8 activates caspase-3 and induces apoptosis [46]. The endogenous pathway is regulated by the Bcl-2 family of proteins, which are composed of proapoptotic factor Bax and antiapoptotic factor Bcl-2. Members of the Bcl-2 family control mitochondrial permeability and thus control the release of cytochrome *c* [47]. Cytochrome *c* activates caspase-9, and then, caspase-3 is also activated, eventually leading to cell apoptosis [48]. In this study, H_2_O_2_ exposure led to increases in the expressions of Fas, cleaved caspase-8, cleaved caspase-3, cleaved caspase-9, and cleaved PARP, as well as increases the Bax/Bcl-2 ratio. At the same time, H_2_O_2_ significantly increased the release of cytochrome *c*. However, phillyrin pretreatment effectively reversed these changes. These results indicate that phillyrin effectively inhibited the activation of endogenous and exogenous apoptotic pathways in H_2_O_2_-stimulated RPE cells. The mechanism of phillyrin protecting RPE cells from H_2_O_2_-induced apoptosis through the Nrf2 signaling pathway is shown in Figure 9.

Oxidative stress causes RPE dysfunction or apoptosis and eventually leads to AMD [49]. Oxidative stress increases ROS in cells [50]. Excessive ROS changes the Bax/Bcl-2 ratio, leading to the formation of channels in the mitochondrial membrane and release of cytochrome *c* into the cytoplasm [51]. Superoxide dismutase (SOD) and GSH are natural antioxidants that scavenge free radicals in cells [52]. In this study, compared with the control group, H_2_O_2_ caused significant increases in intracellular ROS levels and Bax/Bcl-2 ratio and led to decreases in MMP as well as release of cytochrome *c*, while phillyrin pretreatment effectively reversed these changes. In addition, phillyrin pretreatment blocked the H_2_O_2_-induced increases in MDA and ROS levels and increased SOD and GSH levels. Therefore, it can be inferred that phillyrin inhibited the imbalance between oxidation and antioxidation caused by H_2_O_2_ and effectively improved the antioxidant capacity of RPE cells.

Under pathological conditions, the mitochondrial respiratory chain is the main source of intracellular ROS [53]. When ROS are present in high levels, Nrf2 is stimulated, and it dissociates from its complex with Keap1 and is transferred to the nucleus to bind to ARE, leading to activation of the expressions of oxidoreductases such as GCLC/HO-1/NQO-1 [54]. Therefore, the Nrf2 pathway regulate ROS levels, and it is an effective pathway for reduction of oxidative stress-induced damage in cells. In this study, phillyrin significantly inhibited H_2_O_2_-induced decreases in the expressions of total Nrf2 and nuclear Nrf2 in RPE cells. In addition, phillyrin also effectively blocked H_2_O_2_-induced increase in the Keap1/Nrf2 protein complex formation. These results indicate that antioxidant defenses in RPE cells were inhibited by H_2_O_2_, but this effect was effectively reversed by phillyrin through the activation of the Nrf2 pathway. Addition of ML385 to the high-dose phillyrin+H_2_O_2_ group blocked the increase in the protein expression of total Nrf2. This result confirms that phillyrin protected RPE cells from H_2_O_2_-induced oxidative stress damage through the Nrf2 signaling pathway. Moreover, H_2_O_2_ inhibited nuclear translocation of Nrf2, thereby inhibiting the expression of downstream antioxidant proteins. However, pretreatment with phillyrin enhanced the nuclear translocation of Nrf2, leading to a successful activation of the expressions of downstream antioxidant proteins and protection of RPE cells from oxidative damage. Therefore, the antioxidant pathway mediated by the Nrf2 signaling pathway is one of the mechanisms by which phillyrin protected RPE cells from oxidative damage induced by H_2_O_2_.

Studies have shown that DNA damage is closely related to oxidative stress [55]. Oxidative damage caused by excessive ROS activates p53, and it rapidly accumulates in the nucleus [56]. In turn, p53 affects the expressions of Bcl-2 and Bax and ultimately leads to apoptosis [57]. Cyclin, CDK, and cyclin-dependent kinase inhibitors (CDKI) control cell cycle progression [58]. The p21 protein inhibits the expression of CDK [59]. It has been found that p21 blocks the cell cycle progression from the S-phase to the G0/G1 phase by inhibiting cyclin A. In this study, H_2_O_2_ significantly downregulated the expressions of p53, cyclin A, cyclin E, and CDK, while the protein expressions of p-p53 and p21 were upregulated. However, these changes were significantly reversed by phillyrin pretreatment. These results suggest that excessive ROS may cause DNA oxidative damage in RPEs, leading to the G0/G1 phase cell cycle arrest and mitochondria-dependent apoptosis. In effect, these results indicate that phillyrin indirectly alleviated the G0/G1 phase cell cycle arrest and mitochondria-dependent apoptosis by inhibiting the ROS-mediated p53/p21 signaling pathway.

## 5. Conclusion

The results obtained in this study indicate that phillyrin protects RPE cells from oxidative stress-induced apoptosis and G0/G1 cell cycle arrest by activating the Nrf2 signaling pathway. This is the first study showing that phillyrin pretreatment improves the antioxidant capacity of RPE cells. This finding provides new strategies for development of new drugs for the treatment of oxidative stress-related AMD and retinal degenerative diseases.

## Data Availability

The data that support the findings of this study are available from the corresponding author on reasonable request.
