# Erratum to “Phillyrin Mitigates Apoptosis and Oxidative Stress in Hydrogen Peroxide-Treated RPE Cells through Activation of the Nrf2 Signaling Pathway”

**DOI:** 10.1155/2023/9891240

**Published:** 2023-10-11

**Authors:** Yuanyuan Du, Longtai You, Boran Ni, Na Sai, Wenping Wang, Mingyi Sun, Rui Xu, Yu Yao, Zhiqin Zhang, Changhai Qu, Xingbin Yin, Jian Ni

**Affiliations:** ^1^School of Chinese Materia Medica, Beijing University of Chinese Medicine, Beijing 100029, China; ^2^Dongzhimen Hospital Affiliated to Beijing University of Chinese Medicine, Beijing 100029, China; ^3^School of Pharmacy, Inner Mongolia Medical University, 010110 Hohhot, China

In the article titled “Phillyrin Mitigates Apoptosis and Oxidative Stress in Hydrogen Peroxide-Treated RPE Cells through Activation of the Nrf2 Signaling Pathway” [1], there were errors in Figures 2(e), 3(a) and 3(b), 4(a), 5(a) and 5(c), 7(a), and 8(d) where the incorrect units were stated (M vs. *μ*M).

These errors were inadvertently introduced during the production process of the article.

Figure 2(e) should be corrected as follows, and is listed as Figure 1:

Figures 3(a) and 3(b) should be corrected as follows, and is listed as Figure 2:

Figure 4(a) should be corrected as follows, and is listed as Figure 3:

In Figure 5(c), the *β*-actin and mitochondrial cytochrome *c* protein bands are incorrectly duplicated. The corrected figure is shown below, and is listed as Figure 4:

Figure 7(a) should be corrected as follows, and is listed as Figure 5:

Figure 8(d) should be corrected as follows, and is listed as Figure 6:

Additionally, affiliation number 4 was included by error. The correct affiliation list and author affiliations are shown above. The Authors' Contributions section is revised as below:

## Figures and Tables

**Figure 1:**
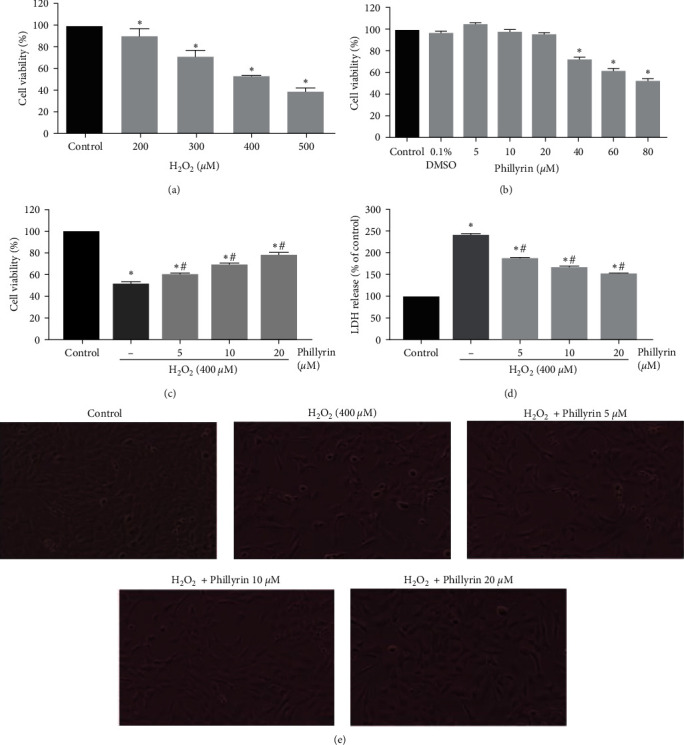
Protective effect of phillyrin on H_2_O_2_-induced cytotoxicity in RPEs. (a) RPE cells were treated with indicated concentrations of H_2_O_2_ for 6 hours, and cell viability was analyzed with the MTT assay. (b) Cell viability of RPE cells treated with different concentrations of phillyrin. (c) RPE cells were pretreated under different concentrations of phillyrin (5, 10, and 20 *μ*M) for 24 hours and then incubated with 400 *μ*M H_2_O_2_ for 6 hours. The cytoprotective effect of phillyrin was measured with the MTT assay. (d) LDH profile showing the dose-dependent cytoprotective effect of phillyrin. (e) Morphological changes in RPE cells (original magnification: ×200). Compared with the H_2_O_2_ group, phillyrin pretreatment protected RPE cells in a dose-dependent manner. Data are expressed as mean ± S.D. Experiments were repeated three times ( ^*∗*^*p* < 0.05 vs. control, #*p* < 0.05 vs. H_2_O_2_-treated group).

**Figure 2:**
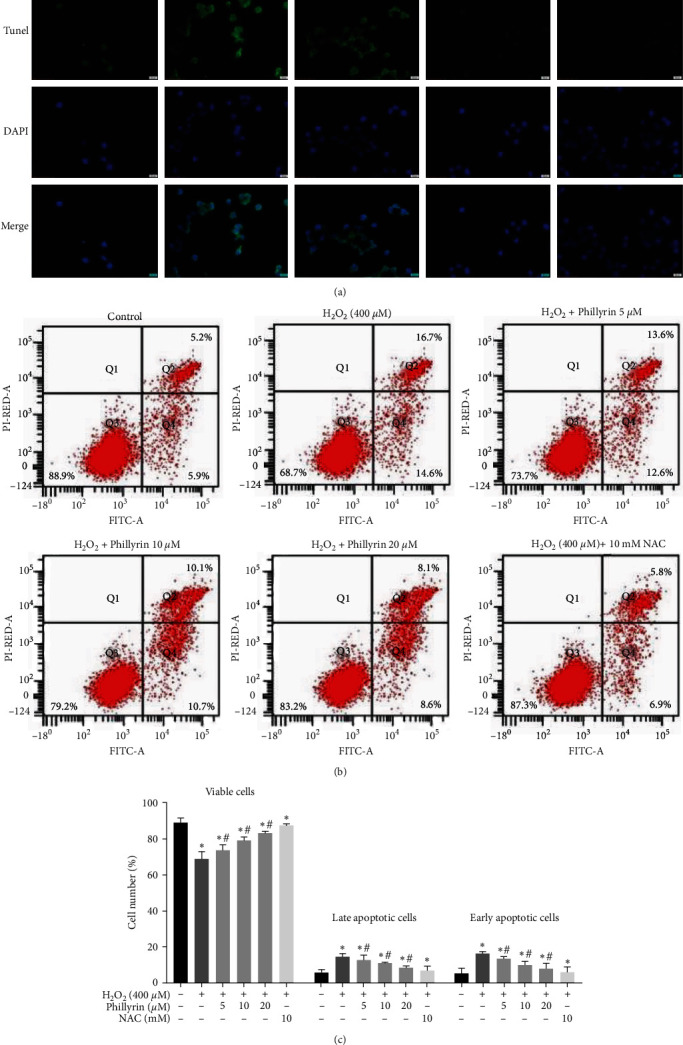
Protective effect of phillyrin on apoptosis of RPE cells. (a) Morphological changes in the nuclei of RPE cells, as revealed via TUNEL staining. (b) Apoptosis of RPE cells treated with different concentrations of phillyrin, as detected using double staining of Annexin V-FITC/PI and flow cytometry. (c) Histogram of average cell fluorescence showing survival, early apoptotic, and late apoptotic cells. Data are expressed as mean ± S.D. Experiments were repeated three times ( ^*∗*^*p* < 0.05 vs. control, #*p* < 0.05 vs. H_2_O_2_-treated group).

**Figure 3:**
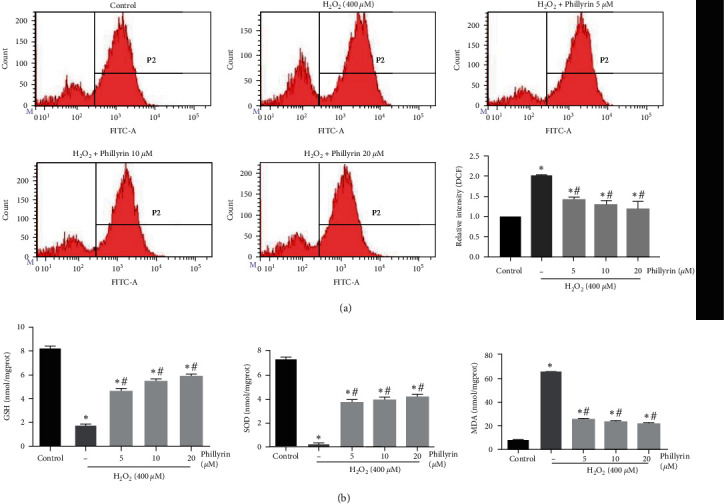
Effect of phillyrin on ROS and the antioxidant enzyme activity in RPE cells after oxidative stress injury. (a) Effect of H_2_O_2_ and different concentrations of phillyrin on ROS, as evaluated using flow cytometry. (b) Effect of H_2_O_2_ and different concentrations of phillyrin on the MDA content and levels of antioxidants SOD and GSH in the cells. Data are expressed as mean ± S.D. Experiments were repeated three times ( ^*∗*^*p* < 0.05 vs. control, #*p* < 0.05 vs. H_2_O_2_-treated group).

**Figure 4:**
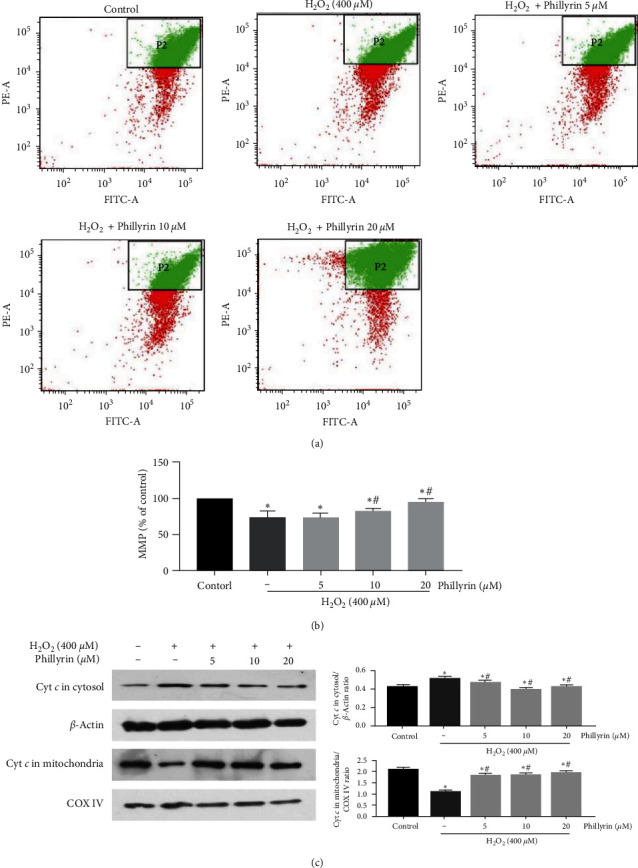
Effect of phillyrin on MMP in H_2_O_2_-treated RPE cells. (a) MMP in the cells after intervention with H_2_O_2_ and different concentrations of phillyrin, as determined using JC-1 staining. (b) Histogram of average cell fluorescence. (c) Effect of different concentrations of phillyrin on the release of cytochrome c in mitochondria and cytoplasm, as measured using Western blotting. The protein bands were quantified with density analysis and statistically analyzed. The internal controls for cytoplasm and mitochondria were *β*-actin and COX IV, respectively. Data are expressed as mean ± S.D. Experiments were repeated three times ( ^*∗*^*p* < 0.05 vs. control, #*p* < 0.05 vs. H_2_O_2_-treated group).

**Figure 5:**
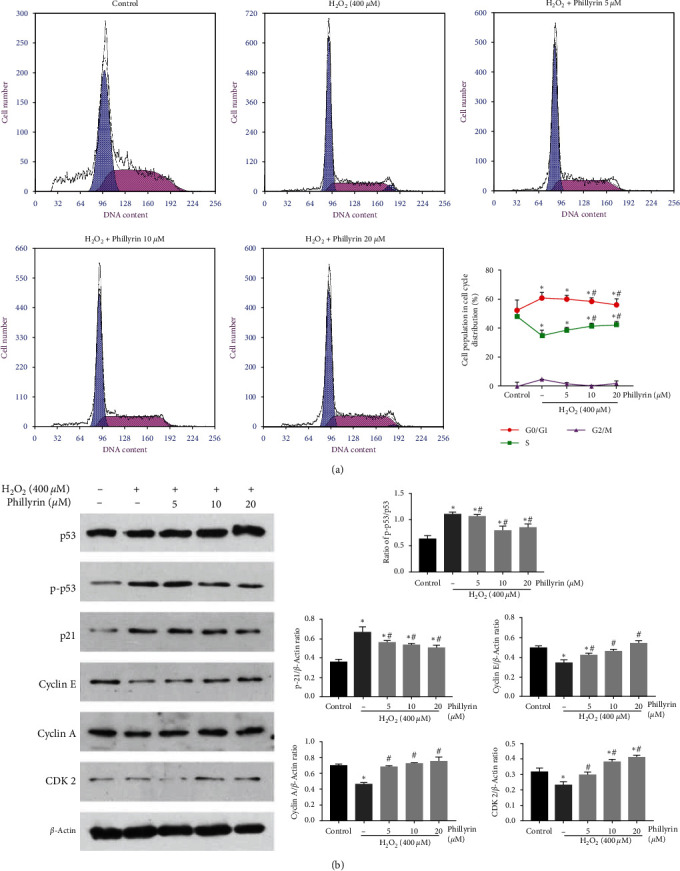
Effect of phillyrin on H_2_O_2_-indued cell cycle arrets in RPE cells. (a) Effect of different concentrations of phillyrin on each stage of the cell cycle. (b) Effect of phillyrin on the expression levels of cell cycle-related proteins, as measured using Western blotting. Data are expressed as mean ± S.D. Experiments were repeated three times ( ^*∗*^*p* < 0.05 vs. control, #*p* < 0.05 vs. H_2_O_2_-treated group).

**Figure 6:**
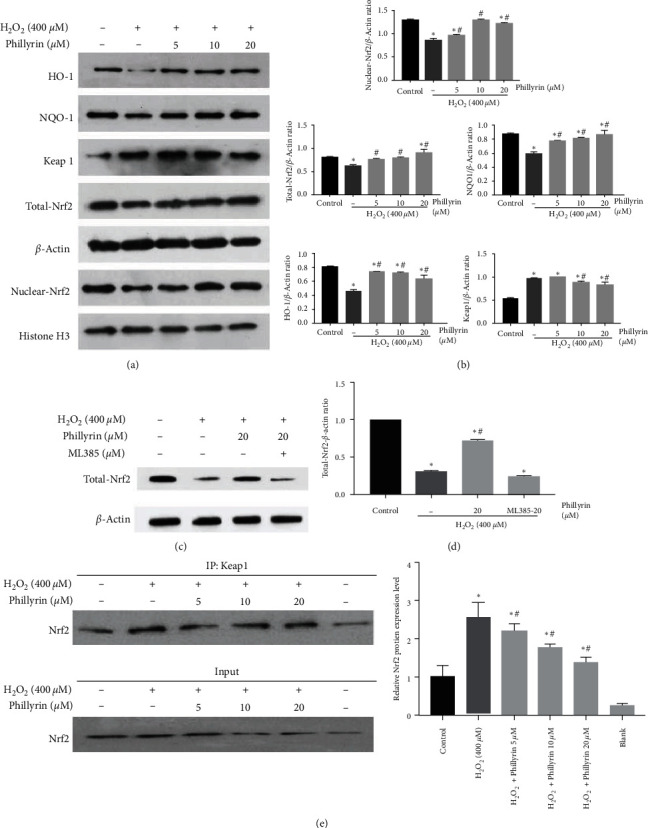
Effect of phillyrin on the Nrf2/HO-1 pathway in H_2_O_2_-treated RPE cells. (a) Expression levels of HO-1, NQO1, Keap1, total Nrf2, and nuclear Nrf2, as assayed using Western blotting. (b) Quantitative analysis of the protein bands using optical density analysis. (c) After adding ML385 to the high-dose phillyrin + H_2_O_2_ group, the expression levels of total Nrf2, as assayed using Western blotting. (d) Quantitative analysis of the protein bands using optical density analysis. (e) Effect of phillyrin on the formation of the Keap1/Nrf2 complex, as detected using the coimmunoprecipitation assay. Data are expressed as mean ± S.D. Experiments were repeated three times ( ^*∗*^*p* < 0.05 vs. control, #*p* < 0.05 vs. H_2_O_2_-treated group).
